# Embrittlement Fracture Behavior and Mechanical Properties in Heat-Affected Zone of Welded Maraging Steel

**DOI:** 10.3390/ma17020440

**Published:** 2024-01-17

**Authors:** Akihiro Takahashi, Toshinobu Toyohiro, Yuji Segawa, Masakazu Kobayashi, Hiromi Miura

**Affiliations:** 1 Department of Mechanical Engineering, National Institute of Technology, Miyakonojo College, Miyazaki 885-8567, Japan; 2 Department of Mechanical Engineering, Toyohashi University of Technology, Toyohashi 441-8580, Japan

**Keywords:** maraging steel, mechanical property, heat-affected zone, embrittlement fracture

## Abstract

In welded maraging steels, mechanical properties, particularly ductility and toughness, are often compromised in the heat-affected zone (HAZ). This study focuses on 300-grade maraging steel bars, solution annealed at 1123 K for 1.5 h (5.4 ks) and welded using gas tungsten arc welding, followed by a post-weld heat treatment at 753 K for 13.33 h (48 ks). In situ observations during three-point bending tests on HAZ samples featuring coarsened prior austenite grain sizes were conducted to examine damage behavior and the crack path near the crack tip. The main crack initiated at the peak applied load during the bending test and, upon further loading, exhibited significant deflection and extension accompanied by numerous microcracks and localized crack branching. Distinctive damage features, such as transgranular cracking across block regions, intense intergranular cracking along packet boundaries with a pronounced shear component, and crowding of microcracks ahead of the crack tip, were observed in the HAZ sample during the in situ test. The interaction between the main crack tip and microcracks and its influence on the local crack propagation driving force was discussed using fracture mechanics. Experimental results, including tensile fracture surface observations and in situ images, along with analysis of the stress anti-shielding effect by microcracks, suggest that the HAZ sample exhibits embrittlement fracture behavior with lower ductility and toughness compared to the base metal sample.

## 1. Introduction

Steel materials can be categorized based on room temperature (RT) tensile strength, σ_B_, into two types: (1) high-strength steel (HSS) [1] and advanced high-strength steel (AHSS) [2,3], used for plates and pipes in civil, building, ship, vessel, and vehicle construction [4,5,6] within general industrial structure sectors, and (2) ultra-high strength steel (UHSS) [1], employed in the manufacture of aircraft, space-rockets, dies, deep-sea submersibles, and centrifugal separators for uranium enrichment [7,8,9,10,11], pertinent to advanced technology sectors. The development of HSSs aims to enhance economic efficiency by minimizing material and construction costs. In contrast, UHSSs necessitate more attention to strength and toughness compared to HSS, leading to a significant increase in material costs. However, UHSSs are essential for achieving the requisite performance in machinery and structures. Thus, UHSSs are utilized for critical applications, though they are not as cost-effective as HSSs. Consequently, UHSS not only represents the strongest category among steel materials, exhibiting a yield strength exceeding 1380 MPa (200 ksi) [12,13], but also features high specific strength and high specific modulus.

Maraging steels, a category of UHSS, have been the subject of extensive research focusing on the interplay between age-hardening, microstructure, mechanical properties, fracture behavior, and heat treatment [14,15,16]. Initially developed by the International Nickel Company Inc. in the 1960s, these steels, based on an Fe–Ni alloy containing significant amounts of Co and Mo with a low carbon content, have evolved over time. Grades of maraging steels, such as 200, 250, 300, 350, and 400/450, are differentiated by their yield strengths in ksi [17]. These steels exhibit a body-centered cubic (BCC) crystal structure and form a low-strength martensite phase after solution annealing treatment. The primary strengthening mechanism in maraging steel is the precipitation of intermetallic compound (IMC) particles during the aging treatment, which impedes the dislocation movement. The appropriate combination of a ductile martensite matrix and a dense array of hard IMCs endows these steels with mechanical properties ideal for applications requiring stringent strength specifications.

Standard heat treatment involves solution annealing above 1073 K followed by air-cooling to RT, resulting in a ductile martensite matrix. Subsequent aging treatment typically occurs between 723 and 823 K, promoting the formation of fine IMC particles such as Ni_3_(Ti, Mo) and Fe_2_Mo within the martensite matrix [18,19,20,21,22]. Prolonged aging has been reported to induce other IMC phases in these steels [23,24]. However, aging can also lead to the formation of retained austenite, either partially or throughout the matrix [25], which influences the steel’s mechanical properties. Ultimately, the hardness and strength of maraging steel are optimized through standard heat treatment, depending on the desired properties.

Since their introduction in the 1960s, maraging steels have garnered significant interest, particularly in the aerospace industry. The research and development concerning higher hardness and strength continues, establishing maraging steel as a key material in this domain. Over the past decade, metal additive manufacturing techniques, notably direct energy deposition and selective laser melting, have been employed to produce maraging steel components [26,27,28,29,30,31,32]. Additionally, ongoing research efforts in the metal manufacturing industry and academia, including precipitate analysis via transmission electron microscopy (TEM) [33,34,35,36] and atom probe tomography [37,38], as well as first-principle calculations and molecular dynamics studies, aim to enhance the RT strength and fatigue properties of maraging steels.

However, welding processes involving high temperatures can diminish the effectiveness of the high hardness and strength of these metals by eliminating fine IMC particles, coarsening the grain size, and introducing other embrittlement factors through microstructural segregation. The weldability of steel, representing its propensity to be welded without defects and the performance of its welded joints in service, is a critical consideration. In the narrower sense, optimal weldability implies welding with minimal brittle heat-affected zones (HAZs) and reduced risk of hydrogen-assisted cold cracking. The weldability of maraging steel has traditionally been assessed based on empirical welding practices for aircraft and rocket components [39,40,41,42]. Welding remains a vital fabrication method for various applications of maraging steel. Several studies have investigated the microstructural evolution in the HAZ of welded maraging steel [43,44,45,46,47,48,49,50,51,52,53,54]. Our experiments [16] demonstrated that the increase in hardness of 300-grade maraging steel is not dependent on its prior austenite grain size. However, extensive grain coarsening significantly reduces ductility and toughness. Moreover, we observed that the hardness distribution in the HAZ adjacent to the fusion metal region remains unchanged despite grain coarsening due to the tungsten inert gas (TIG) arc welding of the maraging steel. This suggests that the deformation capability and toughness of coarsened grains in the HAZ are considerably reduced even at the same hardness level, posing a risk. Thus, welding of maraging steels is an ongoing area of study, with further research needed to fully understand and optimize these processes.

Damage evolution is driven by plastic deformation which leads to the fracture of most metallic materials. Fracture mechanics is a pivotal study field to understand the behavior of metallic materials when subjected to external stresses. Currently, the J integral proposed by Rice [55] is regarded as a major nonlinear fracture mechanics model to characterize crack tip stress fields for power-law hardening materials. Additionally, the stress and strain distributions ahead of the crack tip are obtained by HRR solution [56,57]. Thus, the axial stresses at damaged sites around the crack tip during the growth of the main crack can be calculated by HRR solution. Toda et al. [58,59,60] employed in situ SEM observation and analysis of HRR stress fields to investigate the fracture strength of coarse IMC particles in the vicinity of the crack tip in a wrought aluminum alloy. Based on the HRR solution, Fukumasu et al. [61] calculated the microcrack initiation stress ahead of the crack tip in a sintered γ-base TiAl alloy at high temperature. Microstructural mages during the interrupted fracture toughness test were used to estimate interaction effect between the main crack and microcracks. The crack tip stress shielding effects caused by microcracking have been studied by a number of researchers [62,63,64,65].

The primary objective of this study is to experimentally evaluate the mechanical properties and fracture behavior of the HAZ following TIG welding in maraging steel. To acquire miniature samples with varying prior austenite grain sizes, three-point bending test specimens featuring a V-notch were extracted directly from the HAZ and the base metal areas. Additionally, in situ observation of the bending test was conducted based on an unload–reload examination method. This approach was employed to accurately determine the behavior of plastic deformation and crack propagation near the specimen notch. There is a notable paucity of research focusing on continuous, high-magnification observation to thoroughly understand the fracture behavior in HAZ samples of maraging steel. For comparative purposes, tensile test specimens that were not subjected to welding, possessing identical hardness but different grain sizes, were treated at two different solution annealing temperatures and then tested at RT. The findings of this study can significantly enhance our understanding and application of holistic maraging steel utilization. Furthermore, they can contribute to the advancement of structural design techniques, particularly in relation to weldment products and joint technology.

## 2. Materials and Methods

### 2.1. Experimental Material

**Figure 1:**
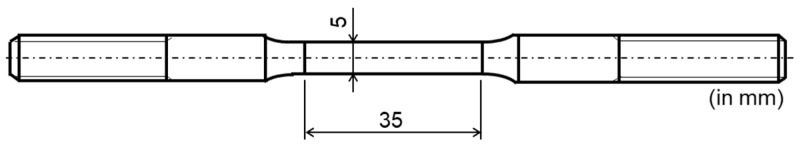
Schematic of the configuration and dimensions of the tensile testing specimen.

The material used in this study was a commercial 18% Ni 300-grade maraging steel provided by Proterial Ltd. (formerly Hitachi Metals Ltd., Tokyo, Japan) in the form of a hot-extruded round bar with a 9 mm diameter, as detailed in Table 1. The as-supplied material was solution-annealed in an inert environment, exhibiting an average Vickers hardness of 307 HV. To obtain samples with varying prior austenite grain sizes, two distinct heat treatments were applied: (a) solution annealing treatment at 1123 K for 1.5 h (5.4 ks), followed by an aging treatment at 753 K for 13.33 h (48 ks) (referred to as STed at 1123 K), and (b) solution annealing treatment at 1373 K for 1 h (3.6 ks), followed by an aging treatment at 753 K for 13.33 h (48 ks) (referred to as STed at 1373 K). These processes were conducted using an electric heating furnace, with all samples being air-cooled to RT. Subsequently, the materials were cut and machined into the dimensions required for tensile testing, as illustrated in Figure 1. The tensile test specimens were machined from the as-received material along the extrusion direction. For digital microscopy analysis, the samples were etched with a 5% nital solution following polishing to a 0.25 μm finish using a diamond paste. The microstructure of each sample’s cross section was then observed.

X-ray diffraction (XRD) measurements were performed on the samples to detect reverted austenite (MoKα). These samples did not form reverted austenite, as reported in a previous study [66]. 

### 2.2. Tensile Test and Hardness Procedure

The tensile testing of specimens was conducted in accordance with Japanese Industrial Standards (JIS) at an initial loading speed of 1.0 mm/min. A universal testing system (AG-Xplus, Shimadzu, Tokyo, Japan) was employed to assess the mechanical properties in this study. Tensile load *F* was measured using a load cell with a capacity of 50 kN. Displacement and tensile strain in the gauge length of the tensile specimen, oriented in the growth direction, were determined using a noncontact extensometer (DVE-101/201, Shimadzu) and a strain gauge (FLK-1-11, Tokyo Measuring Instruments Lab., Tokyo, Japan) [16]. The deformation within the elastic region was evaluated using the strain gauge. For assessing fracture strain beyond the elastic region, the noncontact type of extensometer was utilized. The area measurements for calculating tensile stress (*σ*) and elastic modulus (*E*) were based on the average value of three measured cross-sectional areas (*A*) of the specimen along the longitudinal axis prior to the tensile test. Consequently, the engineering tensile stress was calculated as *σ* = *F*/*A*. The elastic modulus *E* was determined using a regression line fitted to the stress–strain curve in the range of 100 to 300 MPa. A total of five specimens were prepared for the tensile test in this study. Fractographic features were examined using SEM (VHX-D500, Keyence Corp., Itasca, IL, USA). For the detailed interior views during SEM observation, sputter coating with a Pt–Pd target was performed.

The Vickers hardness test (AVK-A, Akashi Seisakusho Ltd., Tokyo, Japan) was conducted in accordance with JIS, using an indenter load of 9.8 N for a duration of 15 s.

### 2.3. TIG Welding and Three-Point Bending Test

**Figure 2:**
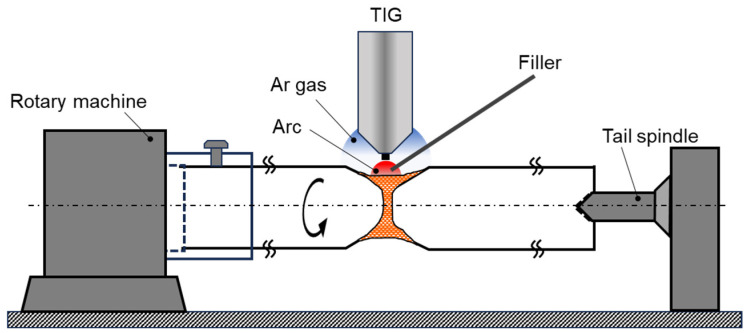
Schematic of the rotary style equipment for full-circle TIG welding.

**Figure 3:**
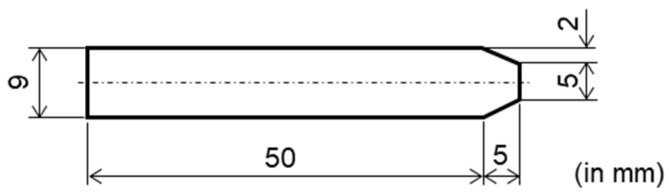
Schematic of the configuration and dimensions of the welding sample.

Butt and full-circle welding experiments were conducted using rotary style equipment (Figure 2) on maraging steel rods that had been STed at 1123 K for 1.5 h (5.4 ks). The dimensions of the maraging steel rod for the welding experiment are shown in Figure 3. The welding joint was completed in three passes using a TIG arc welding setup under an argon gas shield, maintaining a constant current of 35 A and 20 V with a TIG welding machine (YC-300BP4, Panasonic Corp., Tokyo, Japan). The filler used, with a diameter of 1 mm, had the composition as shown in Table 1. The welded joint then underwent a post-weld aging treatment at 753 K, held for 13.33 h (48 ks), followed by air cooling.

**Figure 4:**
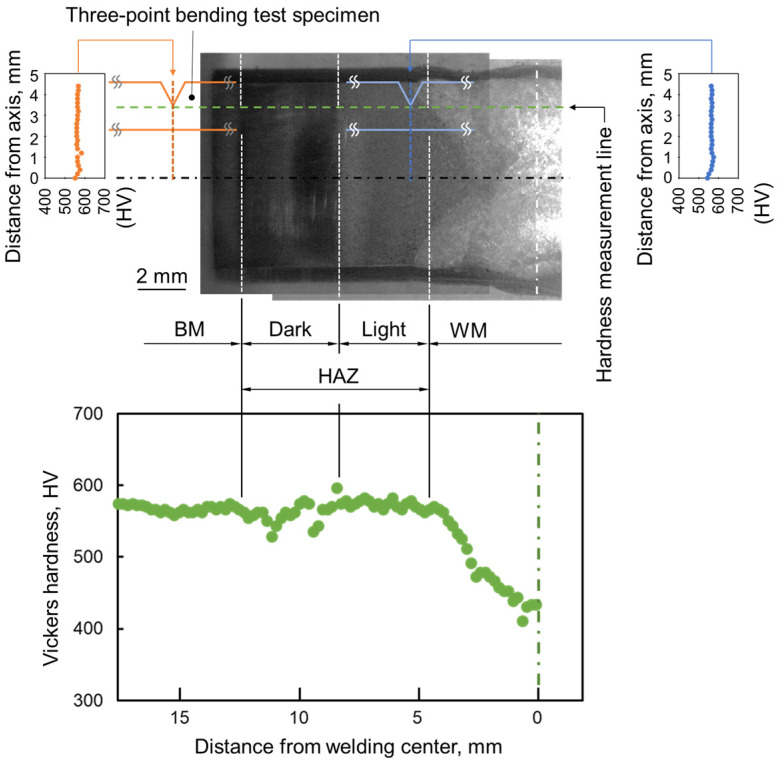
Hardness distribution in the cross section of the post-welded heat-treated joint sample.

For microstructural analysis, the welded joint was polished and etched. Hardness testing was performed using a Vickers hardness tester under the conditions of 9.8 N and 15 s, as previously mentioned. Figure 4 shows the hardness distribution across the cross section of the welded and aged joint sample. Based on the hardness distribution findings, microstructure observations, and other research [51,52], distinct regions such as weld metal (WM), heat-affected zone (HAZ), and base metal (BM) were identified. Miniature three-point bending test specimens with V-notches were extracted from two different regions (as indicated in Figure 4): the BM with a grain size of 10 μm and the HAZ with a grain size of 110 μm, both following a post-weld aging treatment. 

**Figure 5:**
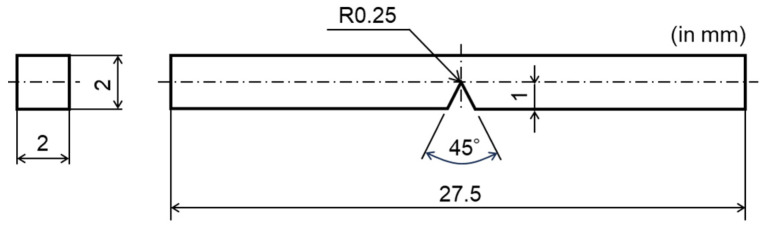
Schematic of the configuration and dimensions of the three-point bending test specimens from the HAZ and BM regions.

Three-point bending specimens of thickness (B) × width (W) × span (S) = 1 mm × 1 mm × 14.6 mm, as shown in Figure 5, were used for in situ observation of the fracture process. These specimens were first mechanically cut from the aforementioned regions (see Figure 4) using a discharge machine and then polished to a 0.25 μm finish using a diamond paste. The fracture experiment for the unload–reload bending test was conducted on a universal testing machine with a capacity of 1 kN (LSC-1/30, Tokyo Koki Testing Machine Corp., Tokyo, Japan) under bending displacement control at a loading speed of 0.05 mm/min at RT. Before the interrupted test, a monotonic three-point bending test was performed continuously at 0.05 mm/min at RT. 

**Figure 6:**
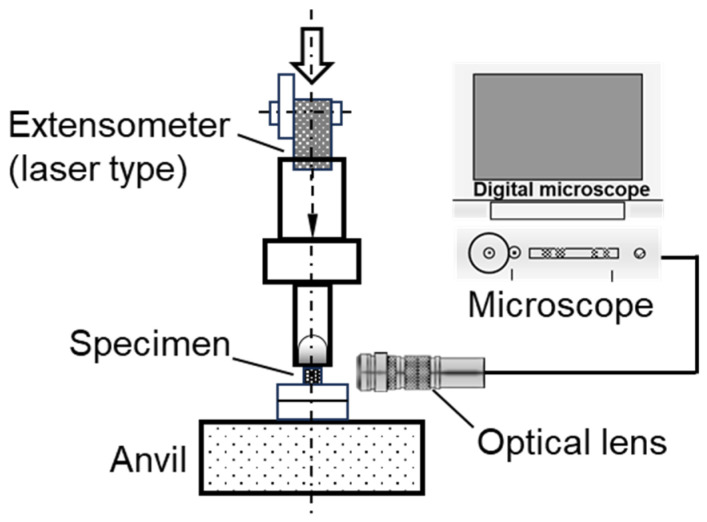
Schematic of the in situ observation setup for the three-point bending test.

Figure 6 shows a schematic of the in situ observation equipment, including a digital microscope (VHX-2000, Keyence Corp.). Load and displacement data were obtained from a load cell and a laser extensometer (IL1000, Keyence Corp.), respectively. A laser point marker, attached to the fixture along the loading line direction, measured the loading line displacement of the specimen. Crack propagation behavior near the V-notch during the unload–reload test was examined, with sequential magnification views captured using the microscope and SEM (VHX-D500, Keyence Corp.).

## 3. Results and Discussion

### 3.1. Microstructure

**Figure 7:**
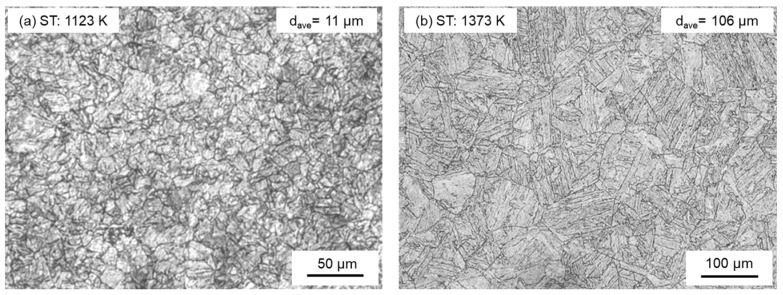
Microstructure of the samples STed at 1123 K and STed at 1373 K. The measured grain sizes in prior austenite are 11 μm in the sample STed at 1123 K (**a**) and 106 μm in the sample STed at 1373 K (**b**).

Figure 7 presents the micrographs of the lath martensite in each sample, etched with 5% nital. The prior austenite grain boundaries in the recrystallized microstructures are distinctly contrasted. The measured grain sizes in prior austenite are 11 μm in the sample STed at 1123 K and 106 μm in the sample STed at 1373 K, determined using the linear intercept method. Typically, dispersoid particles serve as anchors, inhibiting grain growth. At the higher solution annealing temperature of 1373 K, dispersoids diffuse into the grains, resulting in easier spreading of grain boundaries and subsequent coarsening of the grain size. Notably, the martensitic hierarchical microstructure in the sample STed at 1123 K is characteristic of low-carbon steels with prior austenite grains, which are subdivided into packets and blocks composed of laths. These results regarding grain size are consistent with findings reported by other researchers [66,67]. Lima et al. [68] observed that the growth rate of prior austenite grain size is relatively slow between 1133 and 1273 K and increases significantly above 1423 K, with grain size values ranging from approximately 8 μm at 1133 K to 164 μm for the 300-grade maraging steel solution-annealed at 1423 K. Additionally, Lima et al. noted that martensitic plates are more equiaxed up to 1133 K and transform into martensitic blocks at a solution annealing temperature of 1237 K, characterized either by the same orientation or blocks separated by high-angle boundaries [69]. The Vickers hardness values for the samples STed at 1123 and 1373 K are identical in this study, both measuring 561 HV.

### 3.2. Tensile Mechanical Properties

**Figure 8:**
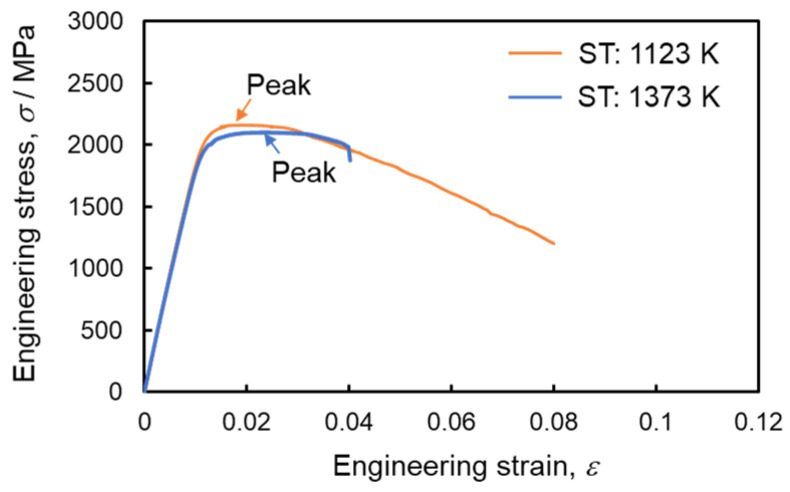
Engineering stress–strain curves of samples STed at 1123 and 1373 K.

The tensile engineering stress–strain curves for samples subjected to two different solution annealing temperatures are depicted in Figure 8. No significant variation was observed in the elastic region, irrespective of the solution annealing temperature, with an elastic modulus of 195 GPa noted in this study. All specimens demonstrated continuous yielding flow behavior. In the sample STed at 1123 K, peak stress was reached early, a characteristic typical of low-carbon martensite steel. This early peak stress in the sample STed at 1123 K is attributed to its high initial mobile dislocation density and a microstructure conducive to cross-slip deformation due to its BCC crystal structure. Consequently, work hardening in this specimen was saturated at an early stage of plastic flow. The tensile-tested specimens exhibited similar mechanical behaviors, with the average ultimate tensile strength (UTS) and 0.2% proof stress for the specimen STed at 1123 K being 2159 MPa and 2113 MPa, respectively; for the specimen STed at 1373 K, these values were 2103 MPa and 2003 MPa, respectively. However, the fracture strain and reduction of area for the specimen STed at 1373 K were 49% and 73% lower, respectively, compared to those for the specimen STed at 1123 K. Thus, the experimental findings of this study suggest that while hardness in 300-grade maraging steel is not dependent on prior austenite grain size, ductility and fracture resistance significantly decrease as the grain size coarsens at higher solution-annealing temperatures. The mechanical properties of the steel specimens subjected to different heat treatments are summarized in Table 2.

**Figure 9:**
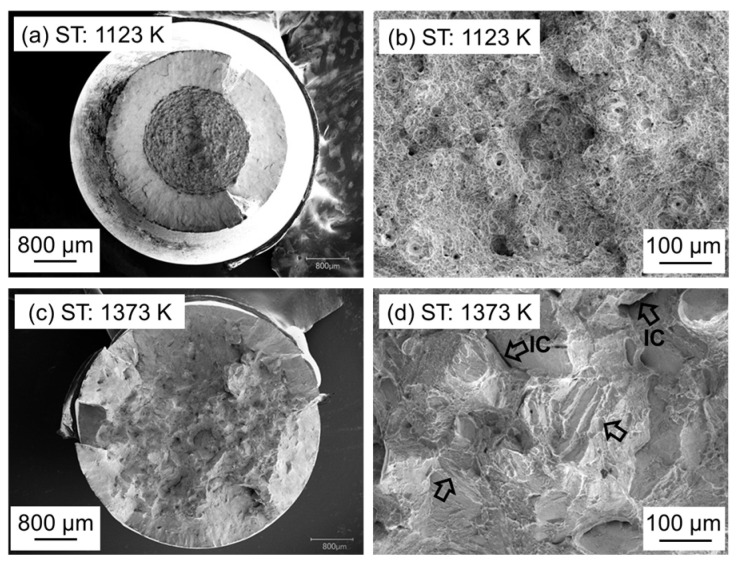
Typical fractographs of the fracture surfaces in samples STed at 1123 and 1373 K after tensile testing. (**a**) shows fracture surface of the specimen STed at 1123 K. (**b**) is a magnified view from the center of (**a**). (**c**) shows fracture surface of the specimen STed at 1373 K. (**d**) is an enlarged photograph from (**c**).

SEM fractography was performed to examine the fracture characteristics of the tensile-tested maraging samples under different solution-annealing temperatures. Figure 9a shows well-proportioned necking deformation and extensive shear-lip rounding on the fracture surface of the specimen STed at 1123 K. Figure 9b is a magnified view from the center of Figure 9a, showing a typical ductile fracture surface with equiaxed dimples, indicative of ductile nucleation, growth, and coalescence of microvoids. The specimen STed at 1373 K exhibited a brittle fracture pattern without necking deformation or shear lips, as seen in Figure 9c. This pattern is consistent with the low reduction in this area of the material. Figure 9d is an enlarged photograph from Figure 9c, showing primarily quasi-cleavage fracture morphologies accompanied by dimples. Notably, distinct intergranular cracks (IC, indicated by arrows) and a decohesion rupture mode, presumably outlining the martensite blocks containing a lamellar structure (as shown by arrows), were also observed. These fracture behaviors, as illustrated in Figure 9, reflect the differences in ductility as observed in the stress–strain curves presented in Figure 8.

### 3.3. In Situ Observation in HAZ and BM

**Figure 10:**
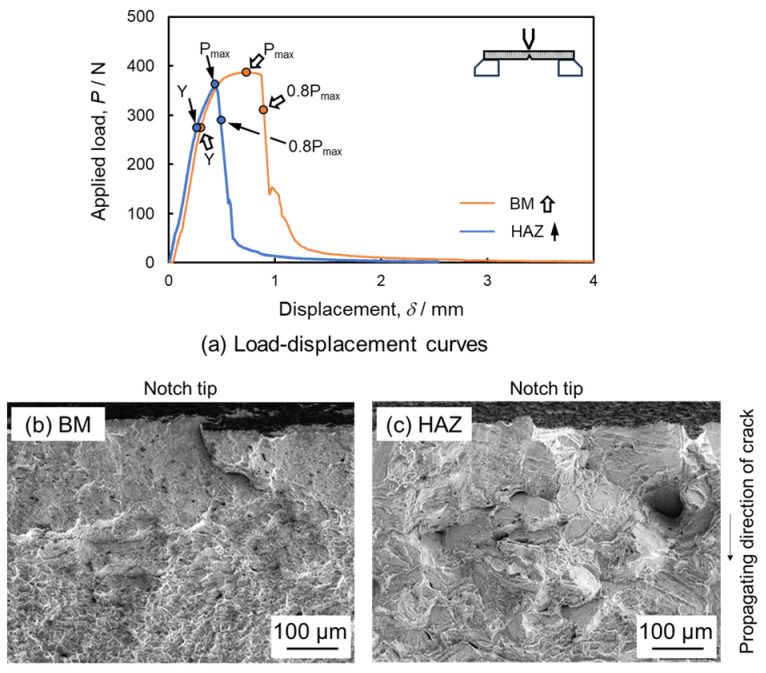
Load–displacement curves (**a**) and fracture surfaces (**b**,**c**) for BM and HAZ samples after three-point bending tests. (**b**) shows fracture surface near the notch tip of the BM specimen. (**c**) shows fracture surface near the notch tip of the HAZ specimen.

The load–displacement curves from the in situ three-point bending tests of the BM and HAZ samples are shown in Figure 10a. Interruptions at various displacements for observation imaging are marked on the curves with symbols such as Y (yield point), P_max_ (peak load), and 0.8P_max_ (0.8 times the peak load past the peak point). Both curves exhibit continuous nonlinear plastic flow beyond the yield point. The peak load and yield point for the BM specimen were 385 and 285 N, respectively, while for the HAZ specimen, they were 358 and 279 N. The peak load in BM is 7% higher than that in HAZ. Figure 4 and Figure 10 collectively suggest that the peak load and Vickers hardness values are similar for both BM and HAZ samples. However, the displacement at peak load and strain energy to failure for the HAZ specimen are 0.44 mm and 127 × 10^−3^ J, respectively, 39% and 58% lower than those for the BM specimen. These findings correlate with the results of tensile properties presented in Figure 8 and Table 2. Figure 10b shows dimples on the fracture surface near the notch tip of the BM specimen. The HAZ specimen displayed a brittle fracture pattern with quasi-cleavage fracture morphologies and intergranular cracks, as seen in Figure 10c. These fracture behaviors reflect the differences in ductility as observed in the load–displacement curves presented in Figure 10a. Moreover, the embrittlement fracture behavior of the HAZ sample is consistent with that of the tensile specimen STed at 1373 K.

**Figure 11:**
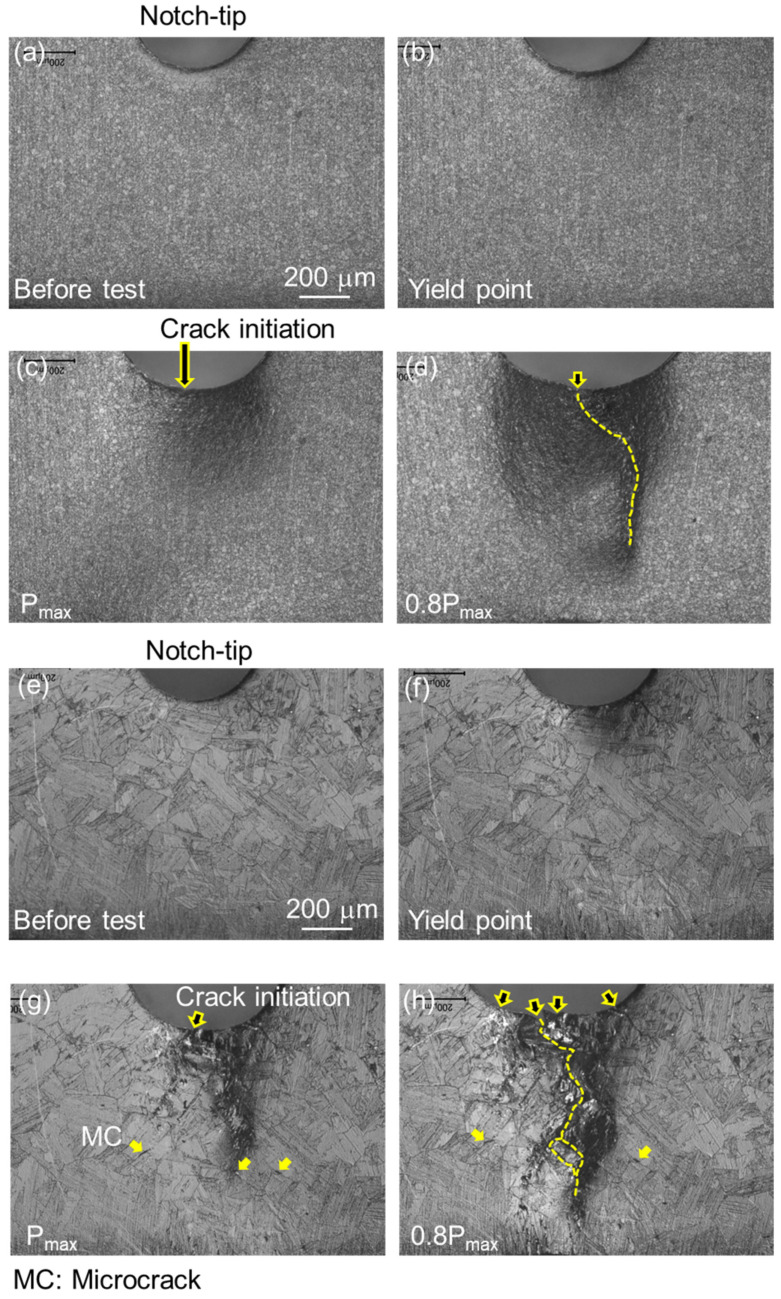
Sequential micrographs of damage behavior near the notch tip in samples BM (**a**–**d**) and HAZ (**e**–**h**), with loading axis (horizontal). These graphs correspond to the loading points indicated Figure 10a, viz., (**a**,**e**) before test; (**b**,**f**) Y point; (**c**,**g**) P_max_; (**d**,**h**) 0.8P_max_.

In situ observation photographs corresponding to each step of the three-point bending test up to the specimen fracture are illustrated in Figure 11. These photographs correspond to the symbols marked on the load–displacement curves in Figure 10a, with the applied loading axis aligned horizontally. Figure 11b shows the microstructure at the yield point of the BM sample. It reveals a plastic strain field extending approximately 160 μm to the right from the notch tip, with no significant damage or microcracks observed near the notch tip. Beyond the yield point, plastic deformation with strain hardening continued, leading to peak load with a plastically strained field of approximately 300 μm and main crack initiation at the notch, as shown in Figure 11c.

Beyond the peak load, the load decreased gradually, followed by a rapid load drop response, concurrent with an increase in main crack opening displacement at the notch edge and expansion of the strain field ahead of the notch tip. Figure 11d depicts the main crack propagation at the point of 0.8P_max_, indicated by a white dashed line. In contrast, the microstructure at the yield point of the HAZ sample (Figure 11f) shows a plastic strain field extending beyond 200 μm and large shear bands near the notch tip. After a further 22% increase in load, the peak load was reached with localized plastic strain and main crack initiation at the notch edge, as shown in Figure 11g. Additionally, microcracks (MC, indicated by yellow arrows) were observed several hundred micrometers from the main crack initiation point. With continued loading, a few large cracks (indicated by arrows) emerged from the notch edge, and a main crack (white dashed line) exhibited significant deflection and extension with crack branching within the localized strain field, as illustrated in Figure 11h.

### 3.4. Crack Propagation Behavior in HAZ

**Figure 12:**
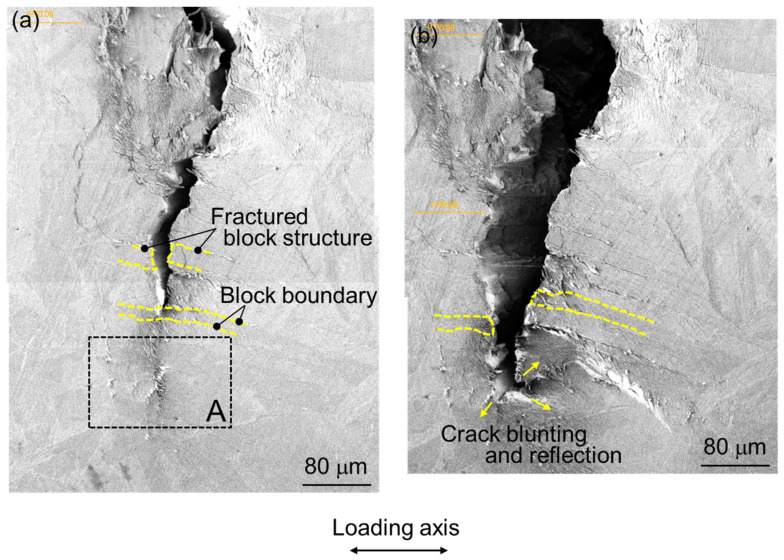
Sequential observations of crack propagation in HAZ sample during three-point bending test. (**a**) shows the photograph of the moment before the main crack tip intersects a block structure within a packet. (**b**) shows crack growth and crack tip blunting as the load increasing.

**Figure 13:**
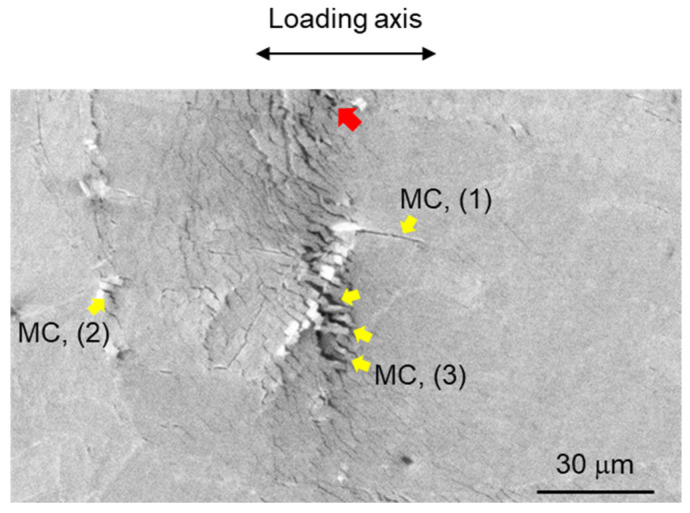
SEM image of section A surrounded by dashed lines in Figure 12a.

The SEM micrographs in Figure 12 illustrate the fracture micromechanisms of crack propagation (specifically transgranular cracking) in the HAZ sample, including numerous microcracks initiated around the crack tip. Figure 12 shows crack propagation in the region of 0.8P_max_ during the three-point bending test. In Figure 12a, the image illustrates the moment before the main crack tip intersects a block structure within a packet. Additionally, secondary cracks, areas of severe localized deformation, and cracks along the boundaries of the block structure are visible in the area where the main crack has already propagated (upper side in crack tip in Figure 12a). Therefore Figure 12a reveals the block structure being intersected by crack propagation, with the crack path discernible through the block structure, showing weak shear deformation. As the load increases, crack growth and crack tip blunting behaviors in three directions are observed, with one direction exhibiting considerable crack deflection while the other two maintain sharp configurations. Their initiations from the blunted crack tip are identifiable in Figure 12b. Figure 13, an enlarged view of section A (outlined by dashed lines in Figure 12a), shows crowding microcracks along the boundaries of laths or sub-blocks in packets, approximately 120 μm ahead of the crack tip. 

Regarding the origins of microcracks in the martensitic microstructure of maraging steels, Fanton et al. [51] suggested the presence of an oxidized layer formed during welding passes. Lima et al. reported the formation of several precipitation particles, such as TiC [69] and Ni_3_Ti [68], at the grain boundary during heat treatment. Viswanathan et al. [18,19] observed that maraging material becomes softer due to the occurrence of the retained austenite phase. Wang et al. [34] noted the presence of both precipitation carbides and a thin austenite phase, with their formation mechanisms analyzed via the Thermo-Cals simulation and other reports [35,70,71,72]. Additionally, thin austenite layers of 5–10 nm thickness and a needle-like shape were detected through TEM and high-resolution TEM experiments. In this study, however, the impact of the retained austenite phase and precipitation particles on the tensile mechanical properties and crack propagation behavior remains unclear. Our next research task involves measuring the austenite phase in the HAZ region using XRD.

Elucidating the effects of microcracks, which serve as precursors to damage, on the macroscopic ductility and fracture toughness of materials is fundamentally important. Understanding the fracture strength of these microcracks, even qualitatively, is vital. Therefore, in situ initiation stresses at microcracks are calculated using a fracture mechanics-based analysis, considering the crack tip singularity field. Because the measured area lies within the plastic zone but outside the intense large-strain region, the strain hardening plasticity solution at a crack tip is applicable for calculating stress distribution in polar coordinates (*r*–*θ*) centered at the crack tip or notch tip. Large deformations would render the HRR theory used in this study invalid; hence, the finite-strain region near the crack tip is excluded from the measurement. The finite-strain region occurs within a distance *r_s_* from the crack tip [73], as expressed in the following equations:

(1) $$r_{s}=\left(e^{\frac{\pi }{2}}-1\right)\cdot \delta_{t},$$

(2) $$\delta_{t}=d_{n}\frac{J}{\sigma_{0}},$$

where $\delta_{t}$ is the crack tip opening displacement, *J* is the J integral, and *d_n_* is a dimensionless constant. This constant is significantly influenced by the strain-hardening exponent and moderately affected by both the flow stress (*σ*_0_) and the elastic modulus (*E*) of the material. Consequently, the large deformation distance, *r_s_*, in the HAZ sample is 53 μm. Therefore, calculations for a microcrack (indicated by a red arrow and located 42 μm from the crack tip as shown in the upper part of Figure 13) are not applicable. The initiation of these microcracks is estimated by calculating axial stresses in the σ_yy_ direction at the positions of the microcracks. In this study, the stress state is analyzed under plane stress conditions, as the main crack propagation behavior and the microcrack observation were focused on the surface of the specimen. The Ramberg–Osgood power–law relationship between plastic strain and stress is given by Equation (3) [74]:

(3) $$\frac{\sigma }{\varepsilon_{y}}=\frac{\sigma }{\sigma_{y}}+\alpha {\left(\frac{\sigma }{\sigma_{y}}\right)}^{n},$$

where α is a dimensionless constant, and *n* is the strain-hardening exponent (n≥1). The values of α and *n* for the sample STed at 1372 K were determined to be 1.0 and 12, respectively, from data obtained in this study. The axial stress *σ_yy_* at initiated microcracks ahead of the main crack was calculated using Equations (4) and (5) [56,57]:

(4) $$\sigma_{ij}=\sigma_{0}{\left(\frac{EJ}{\alpha {\sigma_{0}}^{2}I_{n}r}\right)}^{\frac{1}{n+1}}{\tilde{\sigma }}_{ij}\left(n,\theta \right),$$

(5) $$\sigma_{yy}=\sigma_{rr}{sin}^{2}\theta +\sigma_{\theta \theta }{cos}^{2}\theta +2\sigma_{r\theta }sin\theta cos\theta ,$$

where *J* is the mode *I* stress intensity factor, relevant when crack propagation is interrupted. The distance from the main crack tip is denoted by *r*, *I_n_* is an integration constant dependent on *n*, and ${\tilde{\sigma }}_{ij}$ is a dimensionless function of *n* and *θ*. The terms *I_n_* and ${\tilde{\sigma }}_{ij}$ were determined from reference [75], with *I_n_* being 2.9. An important aspect to consider is the transition of the HRR singularity to a weaker logarithmic form during growth of the main crack [76]. Previous research indicates that this transition occurs when the normalized ductility parameter, $\Omega =E\cdot \varepsilon_{1f}/\sigma_{0}$, exceeds 34.5 [77,78]. In this study, *ε*_1*f*_ is 0.039, resulting in Ω=3.7, confirming that the HRR singularity remains significant even after crack propagation begins. Consequently, the axial stresses *σ*_yy_ for three microcracks labeled (1) to (3) in Figure 13 are 3105, 4321, and 3688 MPa, respectively. This suggests that microcracks are likely to form due to high axial stress, as analyzed through fracture mechanics.

Microcrack shielding, a phenomenon where microcracks near the main crack tip can influence crack propagation, is observed. Generally, the driving force for crack propagation varies significantly based on the proximity and orientation relative to the microcrack, resulting in a local mixed mode at the main crack tip and causing the main crack to deflect. As depicted in Figure 12a,b, the presence of a shear component is observed contributing to the separation of the material, indicating that the main crack propagates in a mixed mode of modes I and II. Toda et al. [79,80,81] discussed the relationship between crack length and k_I_ and k_II_ and the mode I and II driving forces, in a local stress intensity factor at the main crack tip. According to their findings, when a single microcrack is located near the main crack tip and the distance between them is close to half the length of the microcrack, the mode I driving force at the main crack tip increases by 78% due to the influence of the single microcrack. In this study, the main crack is observed to propagate toward the crowded microcracks in the HAZ sample, as shown in Figure 12a, and the merging of the main crack with the microcracks is evident in Figure 12b. This observation implies a stage in the process where the anti-shielding effect of the microcracks becomes significantly pronounced, thereby enhancing the growth of the main crack. Consequently, the propagation of the main crack is drastically accelerated by the presence of microcracks.

**Figure 14:**
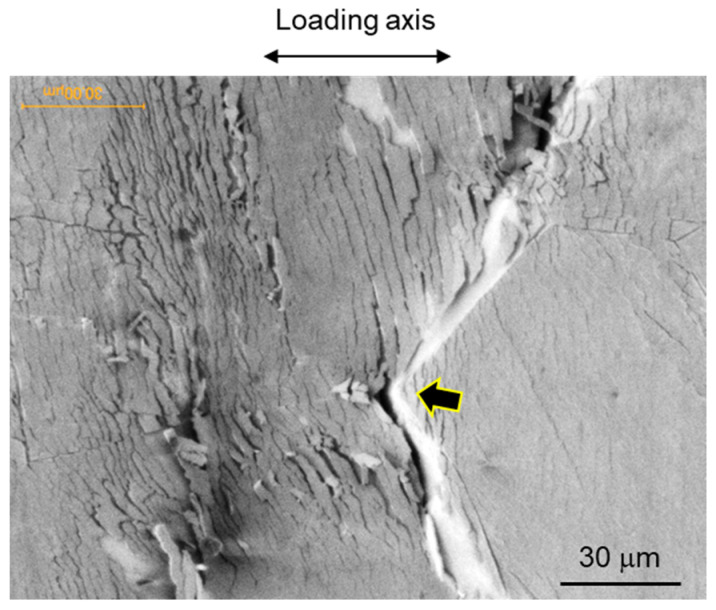
Intense shear cracking at packet boundaries and severe microcracks along the contours of sub-blocks and laths in the HAZ sample. Figure 14 shows observation finding made prior to the crack propagation behavior depicted in Figure 11.

Figure 14 highlights intense shear cracking at packet boundaries and severe microcracks along the contours of sub-blocks and laths in the HAZ sample, corresponding to observations made prior to the crack propagation behavior depicted in Figure 11. Additionally, as indicated in Figure 11g, three microcracks (marked by arrows) are situated ahead of a main crack and occur several hundred micrometers from the main crack tip. Understanding the local interaction of these features and their impact on the overall fracture toughness of maraging steel fabricated through welding necessitates further experimentation and analysis.

The experimental results, including observations of the tensile fracture surface and in situ images, coupled with the analysis of the stress anti-shielding effect by microcracks, suggest that the HAZ sample exhibits lower ductility and toughness compared to the BM sample. Findings in Table 2 and Figure 8 indicate that hardness and tensile strength remain unchanged despite the coarsening of prior austenite grain size due to elevated solution annealing temperatures. The role of prior austenite grain size in strengthening maraging steel remains unclear, given the Hall–Petch relationship, which suggests an ambiguity regarding the influence of block and lath size on strengthening.

This study primarily focused on fracture behavior in the HAZ region, and there are no reports on in situ experiments in the BM sample. Considering the finer prior austenite grain size in the BM region compared to the HAZ, a high-resolution field-emission SEM is required for detailed analysis. A three-point bending test on a specimen STed at 1123 K without a welding history yielded a load–displacement curve almost identical to that of the BM sample in Figure 9. However, future in situ experimentation on the BM sample is necessary for a more accurate understanding of the damage behavior in the HAZ. Moreover, investigation of the WM region, characterized by solidification microstructure and lower hardness than the HAZ and BM, is also desired.

## 4. Conclusions

This study investigated the mechanical properties and fracture behavior of the HAZ in 300-grade maraging steel, TIG welded and subjected to post-weld heat treatment, through the in situ observation of bending tests. Additionally, tensile testing specimens with varying prior austenite grain sizes, treated solely by solution annealing and aging, without a welding history, were prepared for tensile examination. Experimental results by tensile examination suggest that hardness in the prepared samples does not depend on prior austenite grain size, but ductility decreases significantly as grain size coarsens at elevated solution-annealing temperatures. The tensile-tested specimen STed at 1123 K exhibited behavior indicative of typical ductile fracture behavior with equiaxed dimples. On the other hand, the specimen STed at 1373 K exhibited a brittle fracture pattern without necking deformation or shear lips, characterized primarily by quasi-cleavage fracture morphologies. Results from the three-point bending test, hardness distribution and grain size revealed that the peak applied load and Vickers hardness in both BM and HAZ samples were similar. However, the strain energy to failure, or toughness, of the HAZ (grain size of 110 μm) specimen was 127 × 10^−3^ J and 58% lower than that of the BM (grain size of 10 μm) specimen. These measurement findings correlate with the results of tensile properties, hardness, and grain size. In situ observation during the three-point bending test on the HAZ sample revealed significant deflection and extension of the main crack, accompanied by numerous microcracks and localized crack branching. Features such as transgranular cracking across block regions, intense intergranular cracking along packet boundaries with a substantial shear component, and crowding microcracks ahead of the crack tip, were confirmed in the HAZ sample during the test. Thus, the experimental results suggest that the HAZ sample exhibited embrittlement fracture behavior with lower ductility and toughness compared to the BM sample, as evidenced by observations of fracture surfaces after tensile and bending examination.

The advantage of the present investigation is that such in situ fracture examinations make it possible to study and follow deformation behavior on a local scale. In particular, this approach is important to accurately determine, based on fracture mechanics analysis, the behavior of plastic deformation and crack propagation near the crack tip in materials with inhomogeneity microstructures such as weldments, gradient material, castings and composite materials.

## Figures and Tables

**Table 1:** Chemical composition of the 300-grade maraging steel bar and filler used in this study.

Element	C	Ni	Mo	Co	Ti	Al	Si	Mn	Fe
Bar	0.003	18.44	4.89	9.02	0.92	0.11	0.01	0.01	Bal.
Filler	0.002	18.16	4.80	8.54	0.62	0.12	0.04	0.05	Bal.

**Table 2:** Mechanical properties determined by tensile examination in this study.

Solution Annealing (+753 K for 48 ks)	0.2% Proof Stress, *σ*_0.2_, MPa	Ultimate Tensile Strength, *σ_u_*, MPa	Elastic Modulus, *E*, GPa (Strain Gauge)	Fracture Strain, *ε_f_*, (−)	Reduction of Area, *φ*, %
1123 K for 5.4 ks	2113	2159	195	0.079	49.8
1373 K for 3.6 ks	2003	2103	195	0.040	13.2

## Data Availability

Data will be made available upon reasonable request.
